# Supplementary material for: Improved security bound for the round-robin-differential-phase-shift quantum key distribution
Source: Nat Commun. 2018 Jan 31;9:457. doi: 10.1038/s41467-017-02211-x (PMC5792628; doi:10.1038/s41467-017-02211-x)
Supplement: Supplementary file 1 — Supplementary Information [file 41467_2017_2211_MOESM1_ESM.pdf]

SUPPLEMENTARY NOTE 1 - SECURITY PROOF IN THE SINGLE-PHOTON CASE

Alice randomly prepares the single photon state $|\psi\rangle = \sum_{i=1}^{L}(-1)^{k_i}|i\rangle$, where $k_i \in \{0,1\}$ is Alice's raw key bit, and $|i\rangle(i \in \{1,..,L\})$ represents that a single photon is in the i-th time-bin. Eve's general collective attack can be given by:

$$U_{\text{Eve}}|i\rangle|e_{00}\rangle = \sum_{j=1}^{L} c_{ij}|j\rangle\left|e_{ij}\right\rangle \tag{1}$$

where, $\left|e_{ij}\right\rangle$ is the quantum state of Eve's ancilla. Without loss of generality, we assume $c_{ij} \geqslant 0$ and $\sum_{j=1}^{L} c_{ij}^2 \leqslant 1$, where the reason of setting $\sum_{j=1}^{L} c_{ij}^2 \leqslant 1$ is that Eve may introduce vacuum state. For each trial, Eve only retains her ancilla $\left|e_{ij}\right\rangle$ to obtain maximum information on key bits.

In RRDPS protocol, Bob measures the phase shift between $|i\rangle$ and $|j\rangle$ of the incoming single photon states. If Bob projects the incoming single photon states into $(|a\rangle \pm |b\rangle)/\sqrt{2}$ successfully, he will announce $\{a,b\}(a<b)$ to Alice, who will calculate $k_a \oplus k_b$ as her sifted key. The evolution of quantum state is given by

$$\begin{aligned}|\psi\rangle|e_{00}\rangle \longrightarrow &(-1)^{k_a}(\tilde{c}_{aa}|a\rangle + \tilde{c}_{ab}|b\rangle) + (-1)^{k_b}(\tilde{c}_{bb}|b\rangle + \tilde{c}_{ba}|a\rangle) \\ &+ \sum_{i\neq a,b}(-1)^{k_i}(\tilde{c}_{ia}|a\rangle + \tilde{c}_{ib}|b\rangle),\end{aligned} \tag{2}$$

where, $\tilde{c}_{ij} \triangleq c_{ij}\left|e_{ij}\right\rangle$.

The density matrix (non-normallized) of Eve's ancilla will be

$$\begin{aligned}\rho_{\text{E}} &= P\{\frac{1}{\sqrt{2}}((\langle a| + \langle b|)U_{\text{Eve}}|\phi\rangle|e_{00}\rangle\} + P\{\frac{1}{\sqrt{2}}((\langle a| - \langle b|)U_{\text{Eve}}|\phi\rangle|e_{00}\rangle\} \\ &= P\{\sum_{i=1}^{L}(-1)^{k_i}\tilde{c}_{ia}\} + P\{\sum_{i=1}^{L}(-1)^{k_i}\tilde{c}_{ib}\},\end{aligned} \tag{3}$$

where $P\{|x\rangle\} = |x\rangle\langle x|$. Eve aims to guess $k_a \oplus k_b$ after Bob reveals the values of a and b.

Next, we try to simplify Eve's density matrix. Since $k_i(i \neq a,b)$ equals to $0,1$ randomly, the relative phase between $|e_{aa}\rangle(|e_{bb}\rangle)$ and $|e_{ia}\rangle(|e_{bb}\rangle), i \neq a,b$, will be randomized. In other words, we have the following consideration

$$\begin{aligned}\rho_{\text{E}} &\longrightarrow \sum_{j\neq a,b}\sum_{k_j=0,1}\rho_{\text{E}} \\ &= P\{(-1)^{k_a}\tilde{c}_{aa} + (-1)^{k_b}\tilde{c}_{ba}\} + P\{(-1)^{k_b}\tilde{c}_{bb} + (-1)^{k_a}\tilde{c}_{ab}\} \\ &+ \sum_{i\neq a,b} c_{ia}^2 P\{|e_{ia}\rangle\} + c_{ib}^2 P\{|e_{ib}\rangle\}.\end{aligned} \tag{4}$$

Based on the above equation, if $k_a + k_b = 0$, the density matrix (non-normalized) of Eve's ancilla $|e\rangle$ will be

$$\rho_0^{(a,b)} = P\{\tilde{c}_{aa} + \tilde{c}_{ba}\} + P\{\tilde{c}_{bb} + \tilde{c}_{ab}\} + \sum_{i\neq a,b} c_{ia}^2 P\{|e_{ia}\rangle\} + c_{ib}^2 P\{|e_{ib}\rangle\}. \tag{5}$$

If $k_a + k_b = 1$, the density matrix of Eve's ancilla $|e\rangle$ will be

$$\rho_1^{(a,b)} = P\{\tilde{c}_{aa} - \tilde{c}_{ba}\} + P\{\tilde{c}_{bb} - \tilde{c}_{ab}\} + \sum_{i \neq a,b} c_{ia}^2 P\{|e_{ia}\rangle\} + c_{ib}^2 P\{|e_{ib}\rangle\}. \tag{6}$$

Without compromising the security, we can assume that $\langle e_{im}|e_{jn}\rangle = \delta_{ij}\delta_{mn}$. Then, Eve's information on $k_a \oplus k_b$ is given by the Holevo bound [1], which is

$$\begin{aligned} Q^{(a,b)} I_{\mathrm{AE}}^{(a,b)} &\leqslant (c_{aa}^2 + c_{ba}^2) S(\begin{bmatrix} \frac{c_{aa}^2}{c_{aa}^2+c_{ba}^2} & 0 \\ 0 & \frac{c_{ba}^2}{c_{aa}^2+c_{ba}^2} \end{bmatrix}) + (c_{bb}^2 + c_{ab}^2) S(\begin{bmatrix} \frac{c_{bb}^2}{c_{bb}^2+c_{ab}^2} & 0 \\ 0 & \frac{c_{ab}^2}{c_{bb}^2+c_{ab}^2} \end{bmatrix}) \\ &= \varphi(c_{ba}^2, c_{aa}^2) + \varphi(c_{ab}^2, c_{bb}^2), \end{aligned} \tag{7}$$

where, $Q^{(a,b)} = \sum_i (c_{ia}^2 + c_{ib}^2)$ is the yield for any a, b, $\varphi(x^2, y^2) = -x^2 \log_2 x^2 - y^2 \log_2 y^2 + (x^2 + y^2) \log_2 (x^2 + y^2)$. Thus Eve's information on raw key bit is

$$I_{\mathrm{AE}} = \frac{\sum_{a<b} Q^{(a,b)} I_{\mathrm{AE}}^{(a,b)}}{\sum_{a<b} Q^{(a,b)}} \leqslant \frac{\sum_{a<b} \varphi(c_{ba}^2, c_{aa}^2) + \varphi(c_{ab}^2, c_{bb}^2)}{(L-1)\sum_{i,j} c_{ij}^2}. \tag{8}$$

Note that $\varphi(x^2, y^2)$ is a concave function, then using the Jensen's inequality, we have

$$\begin{aligned} \sum_{a<b} \varphi(c_{ba}^2, c_{aa}^2) + \varphi(c_{ab}^2, c_{bb}^2) &\leq \varphi(\sum_{a<b} c_{aa}^2 + c_{bb}^2, \sum_{a<b} c_{ba}^2 + c_{ab}^2)) = \varphi((L-1)\sum_i c_{ii}^2, \sum_{i \neq j} c_{ij}^2) \\ &= \varphi((L-1)x_1, x_2), \end{aligned} \tag{9}$$

where, we define $x_1 = \sum_i c_{ii}^2$ and $x_2 = \sum_{i \neq j} c_{ij}^2$. Consequently, we have

$$I_{\mathrm{AE}} \leqslant \frac{\varphi((L-1)x_1, x_2)}{(L-1)(x_1 + x_2)}. \tag{10}$$

By searching the maximum of above function with free non-negative variables x_1 and x_2 ($x_1 + x_2 > 0$), we can obtain the maximal information leaked to Eve. Next, we try to bound I_{AE} further tightly by finding the relationship between x_1 and x_2.

Intuitively, the parameters x_1 and x_2 may depend on the error rate of the sifted key bit. In the following, we try to introduce the error rate into the security proof of RRDPS protocol. According to Eq.(2), when $k_a + k_b = 0$, the probability of Bob obtaining an error bit is

$$p_e^{(a,b)} = \frac{1}{2}[\left|\tilde{c}_{aa} + \tilde{c}_{ba} - \tilde{c}_{ab} - \tilde{c}_{bb}\right|^2 + \sum_{i \neq a,b} \left|\tilde{c}_{ia} - \tilde{c}_{ib}\right|^2]. \tag{11}$$

In the case that $k_a + k_b = 1$, the probability of Bob obtaining $(|a\rangle + |b\rangle)/\sqrt{2}$ is

$$p_e'^{(a,b)} = \frac{1}{2}[\left|\tilde{c}_{aa} - \tilde{c}_{ba} + \tilde{c}_{ab} - \tilde{c}_{bb}\right|^2 + \sum_{i \neq a,b} \left|\tilde{c}_{ia} + \tilde{c}_{ib}\right|^2]. \tag{12}$$

We are ready to give the relation between error rate $E^{(a,b)}$ and c_{ij}, which is given by

$$
\begin{aligned}
E^{(a,b)} &= \frac{p_e^{(a,b)} + p_e'^{(a,b)}}{Q^{(a,b)}} \\
&= \frac{\left|\tilde{c}_{aa} - \tilde{c}_{bb}\right|^2 + \left|\tilde{c}_{ba} - \tilde{c}_{ab}\right|^2 + \sum_{i \neq a,b} c_{ia}^2 + c_{ib}^2}{2(\sum_i c_{ia}^2 + c_{ib}^2)}.
\end{aligned} \tag{13}
$$

Furthermore, the error for all sifted key bits is

$$
\begin{aligned}
E = \frac{\sum_{a<b} Q^{(a,b)} E^{(a,b)}}{\sum_{a,b} Q^{(a,b)}} &= \frac{\sum_{a<b} \left|\tilde{c}_{aa} - \tilde{c}_{bb}\right|^2 + \left|\tilde{c}_{ba} - \tilde{c}_{ab}\right|^2 + \sum_{i \neq a,b} c_{ia}^2 + c_{ib}^2}{2 \sum_{a<b} \sum_i (c_{ia}^2 + c_{ib}^2)} \\
\geqslant \frac{\sum_{a<b} \sum_{i \neq a,b} c_{ia}^2 + c_{ib}^2}{2 \sum_{a<b} \sum_i (c_{ia}^2 + c_{ib}^2)} &= \frac{(L-2) \sum_{i \neq j} c_{ij}^2 / 2}{(L-1)(\sum_i c_{ii}^2 + \sum_{i \neq j} c_{ij}^2)} = \frac{(L-2) x_2 / 2}{(L-1)(x_1 + x_2)}.
\end{aligned} \tag{14}
$$

Thus, we have $x_2/(x_1+x_2) \leqslant 2(L-1)E/(L-2)$. In conclusion, with this relation, we can calculate a more tighter bound of I_{AE} with (10).

SUPPLEMENTARY NOTE 2 - SECURITY PROOF IN THE TWO-PHOTON CASE

Alice randomly prepares the two-photon state $|\psi\rangle = \sum_{i=1}^{L} |ii\rangle + \sqrt{2} \sum_{1 \leqslant i < j \leqslant L} (-1)^{k_i + k_j} |ij\rangle$, where $k_i, k_j \in \{0, 1\}$ is Alice's raw key bit, and $|ij\rangle (i \in \{1, .., L\})$ represents that there is one photon in the i-th and j-th time-bins respectively. Similar to the single photon case, Eve's general collective attack in two-photon case can be given by:

$$
U_{\mathrm{Eve}} |ij\rangle |e_{000}\rangle = \sum_{l=1}^{L} c_{ijl} |l\rangle |e_{ijl}\rangle. \tag{15}
$$

When Bob projects the incoming single photon states into $(|a\rangle \pm |b\rangle)/\sqrt{2}$ successfully, the evolution of quantum state will be

$$
\begin{aligned}
|\psi\rangle |e_{00}\rangle \longrightarrow & \sum_i \tilde{c}_{iia} |a\rangle + \tilde{c}_{iib} |b\rangle + (-1)^{k_a + k_b} \sqrt{2} (\tilde{c}_{aba} |a\rangle + \tilde{c}_{abb} |b\rangle) \\
& + \sum_{i \neq a,b} (-1)^{k_i} \sqrt{2} ((-1)^{k_a} (\tilde{c}_{iaa} |a\rangle + \tilde{c}_{iab} |b\rangle) + (-1)^{k_b} (\tilde{c}_{iba} |a\rangle + \tilde{c}_{ibb} |b\rangle)) \\
& + \sum_{i<j, i,j \neq a,b} \sqrt{2} (-1)^{k_i + k_j} (\tilde{c}_{ija} |a\rangle + \tilde{c}_{ijb} |b\rangle).
\end{aligned} \tag{16}
$$

For the ease of presentation, we denote $c_{ijl} \left| e_{ijl} \right\rangle$ as $\tilde{c}_{ijl}$, and if $i > j$ for some c_{ijl}, we should recognize it as c_{jil}. For $\sum_i \tilde{c}_{iil}$, we further simplify it as $\tilde{c}_l$. Clearly, as a result of the random phase $(-1)^{k_i}, i \neq a, b$, Eve's state collapses into a mixture state given by

$$
\begin{aligned}
\rho^{(a,b)} = & P\{\tilde{c}_a + (-1)^{k_a + k_b} \sqrt{2} \tilde{c}_{aba}\} + P\{\tilde{c}_b + (-1)^{k_a + k_b} \sqrt{2} \tilde{c}_{abb}\} \\
& + 2 \sum_{i \neq a,b} P\{\tilde{c}_{iaa} + (-1)^{k_a + k_b} \tilde{c}_{iba}\} + P\{\tilde{c}_{ibb} + (-1)^{k_a + k_b} \tilde{c}_{iab}\} \\
& + 2 \sum_{i<j, i,j \neq a,b} P\{\tilde{c}_{ija}\} + P\{\tilde{c}_{ijb}\}.
\end{aligned} \tag{17}
$$

Now based on very similar considerations in single photon case, we write Eve's information as

$$Q^{(a,b)}I_{\mathrm{AE}}^{(a,b)} \leqslant \varphi(\left|\tilde{c}_a\right|^2+\left|\tilde{c}_b\right|^2, 2c_{aba}^2+2c_{abb}^2)+\varphi(2\sum_{i\neq a,b}c_{iaa}^2+c_{ibb}^2, 2\sum_{i\neq a,b}c_{iba}^2+c_{iab}^2). \tag{18}$$

Furthermore, we have

$$\begin{aligned} I_{\mathrm{AE}} =& \frac{\sum_{a<b}Q^{(a,b)}I_{\mathrm{AE}}^{(a,b)}}{\sum_{a<b}Q^{(a,b)}} \\ \leqslant& \frac{\varphi(\sum_{a<b}\left|\tilde{c}_a\right|^2+\left|\tilde{c}_b\right|^2, \sum_{a<b}2c_{aba}^2+2c_{abb}^2)+\varphi(\sum_{a<b}2\sum_{i\neq a,b}c_{iaa}^2+c_{ibb}^2, \sum_{a<b}2\sum_{i\neq a,b}c_{iba}^2+c_{iab}^2)}{\sum_{a<b}\left|\tilde{c}_a\right|^2+\left|\tilde{c}_b\right|^2+2\sum_{i<j}c_{ija}^2+c_{ijb}^2} \\ =& \frac{\varphi((L-1)x_1, x_2)+\varphi((L-2)x_2, 2x_3)}{(L-1)(x_1+x_2+x_3)}, \end{aligned} \tag{19}$$

where,

$$\begin{aligned} x_1 &\triangleq \sum_i \left|\tilde{c}_i\right|^2, \\ x_2 &\triangleq \sum_{a<b}2c_{aba}^2+2c_{abb}^2, \\ x_3 &\triangleq \sum_{a<b}\sum_{i\neq a,b}2c_{abi}^2. \end{aligned} \tag{20}$$

Here, to obtain (19) we used the Jensen's inequality and the following mathematical observations:

$$\begin{aligned} &\sum_{a<b}\left|\tilde{c}_a\right|^2+\left|\tilde{c}_b\right|^2=(L-1)x_1, \\ &\sum_{a<b}\sum_{i\neq a,b}2c_{iaa}^2+2c_{ibb}^2=(L-2)x_2, \\ &\sum_{a<b}\sum_{i\neq a,b}2c_{iab}^2+2c_{iba}^2=2x_3, \end{aligned} \tag{21}$$

hold for any non-negative array. Next we try to analyze the restrictions on x_1, x_2 and x_3 with the help of error rate E.

We return to Eq.(16), it's straightforward to see that the probability for error key events from the first row of Eq.(16) is

$$\begin{aligned} &\frac{1}{2}\sum_{a<b}\left|\tilde{c}_a-\tilde{c}_b+\sqrt{2}(\tilde{c}_{aba}-\tilde{c}_{abb})\right|^2+\left|\tilde{c}_a+\tilde{c}_b-\sqrt{2}(\tilde{c}_{aba}+\tilde{c}_{abb})\right|^2 \\ &\geqslant \sum_{a<b}(\sqrt{\left|\tilde{c}_a\right|^2+\left|\tilde{c}_b\right|^2}-\sqrt{2c_{aba}^2+2c_{abb}^2})^2 \\ &\geqslant (\sqrt{\sum_{a<b}\left|\tilde{c}_a\right|^2+\left|\tilde{c}_b\right|^2}-\sqrt{\sum_{a<b}2c_{aba}^2+2c_{abb}^2})^2, \\ &= (\sqrt{(L-1)x_1}-\sqrt{x_2})^2 \end{aligned} \tag{22}$$

where, we used the Cauchy-Schwartz inequality twice. And the probability for error-key events from the

third row of Eq.(16) is

$$\frac{1}{2}\sum_{a<b}\sum_{i,j\neq a,b}2(c_{ija}^2+c_{ijb}^2)=\frac{(L-3)x_3}{2}. \tag{23}$$

Summing over the Eq.(22) and (23), dividing by $\sum_{a<b}Q^{(a,b)}$, we have

$$E\geqslant\frac{(\sqrt{(L-1)x_1}-\sqrt{x_2})^2+(L-3)x_3/2}{(L-1)(x_1+x_2+x_3)}. \tag{24}$$

This ends the analyses on two-photon case.

SUPPLEMENTARY NOTE 3 - SECURITY PROOF IN THE THREE-PHOTON CASE

Alice randomly prepares the three-photon state

$$|\psi\rangle=\sum_{i=1}^{L}(-1)^{k_i}(|iii\rangle+\sqrt{3}\sum_{j\neq i}|ijj\rangle)+\sqrt{6}\sum_{1\leqslant i<j<l\leqslant L}(-1)^{k_i+k_j+k_l}|ijl\rangle, \tag{25}$$

where $k_i,k_j,k_l\in\{0,1\}$ are Alice's raw key bit, and $|ijl\rangle(i,j,l\in\{1,..,L\})$ represents that there is one photon in the i-th, j-th and l-th time-bins respectively. Similar to the single photon case, Eve's general collective attack in three-photon case can be given by:

$$U_{\rm Eve}|ijl\rangle|e_{0000}\rangle=\sum_{t=1}^{L}c_{ijlt}|t\rangle\big|e_{ijlt}\big\rangle. \tag{26}$$

When Bob projects the incoming single photon states into $(|a\rangle\pm|b\rangle)/\sqrt{2}$ successfully, the evolution of quantum state will be

$$\begin{aligned}|\psi\rangle|e_{0000}\rangle\longrightarrow&(-1)^{k_a}(\tilde{c}_{aa}|a\rangle+\tilde{c}_{ab}|b\rangle)+(-1)^{k_b}(\tilde{c}_{bb}|b\rangle+\tilde{c}_{ba}|a\rangle)\\&+\sum_{i\neq a,b}(-1)^{k_i}(\tilde{c}_{ia}|a\rangle+\tilde{c}_{ib}|b\rangle+\sqrt{6}(-1)^{k_a+k_b}(\tilde{c}_{iaba}|a\rangle+\tilde{c}_{iabb}|b\rangle))\\&+\sum_{i,j\neq a,b}(-1)^{k_i+k_j}\sqrt{6}((-1)^{k_a}(\tilde{c}_{ijaa}|a\rangle+\tilde{c}_{ijab}|b\rangle)+(-1)^{k_b}(\tilde{c}_{ijba}|a\rangle+\tilde{c}_{ijbb}|b\rangle))\\&+\sum_{i,j,l\neq a,b}(-1)^{k_i+k_j+k_l}\sqrt{6}(\tilde{c}_{ijla}|a\rangle+\tilde{c}_{ijlb}|b\rangle),\end{aligned} \tag{27}$$

where, $\tilde{c}_{ij}\triangleq\tilde{c}_{iiij}+\sqrt{3}\sum_{t\neq i}\tilde{c}_{ittj}$, and $\tilde{c}_{ijlt}\triangleq c_{ijlt}\big|e_{ijlt}\big\rangle$. We have observed that its first row and third row have very similar same form with the evolution of single photon given by Eq.(2), while the second row has the similar form with the first row of Eq.(16). Thus, analogous to the calculations in single photon and two-photon cases, we have

$$I_{\rm AE}=\frac{\sum_{a<b}Q^{(a,b)}I_{\rm AE}^{(a,b)}}{\sum_{a<b}Q^{(a,b)}}=\frac{\varphi((L-1)x_1,x_2)+\varphi((L-2)x_2,2x_3)+\varphi((L-3)x_3,3x_4)}{(L-1)(x_1+x_2+x_3+x_4)}, \tag{28}$$

where,

$$
\begin{aligned}
x_1 &\triangleq \sum_i \left|\tilde{c}_{ii}\right|^2,\\
x_2 &\triangleq \sum_{i\neq j} \left|\tilde{c}_{ij}\right|^2,\\
x_3 &\triangleq \sum_{i<j<l} 6c_{ijli}^2 + 6c_{ijlj}^2 + 6c_{ijll}^2,\\
x_4 &\triangleq \sum_{i<j<l}\sum_{t\neq i,j,l} 6c_{ijlt}^2.
\end{aligned} \tag{29}
$$

Here, to obtain (28) we used the Jensen's inequality and the following mathematical observations:

$$
\begin{aligned}
&\sum_{a<b} \left|\tilde{c}_{aa}\right|^2 + \left|\tilde{c}_{bb}\right|^2 = (L-1)x_1,\\
&\sum_{a<b}\sum_{i\neq a,b} \left|\tilde{c}_{ia}\right|^2 + \left|\tilde{c}_{ib}\right|^2 = (L-2)x_2,\\
&\sum_{a<b}\sum_{i,j\neq a,b} 6c_{ijaa}^2 + 6c_{ijbb}^2 = (L-3)x_3,\\
&\sum_{a<b}\sum_{i\neq a,b} 6c_{iaba}^2 + 6c_{iabb}^2 = 2x_3\\
&\sum_{a<b}\sum_{i,j\neq a,b} 6c_{ijab}^2 + 6c_{ijba}^2 = 3x_4,
\end{aligned} \tag{30}
$$

hold for any non-negative array. Next we try to analyze the restrictions on x_1, x_2 and x_3 with the help of error rate E. Based on similar method in last subsection, we have

$$
E \geqslant \frac{(\sqrt{(L-2)x_2} - \sqrt{2x_3})^2 + (L-4)x_4/2}{(L-1)(x_1+x_2+x_3+x_4)}, \tag{31}
$$

where, we used Cauchy-Schwartz inequality and the following mathematical identity

$$
\sum_{a<b}\sum_{i<j<l,i,j,l\neq a,b} 6c_{ijla}^2 + 6c_{ijlb}^2 = (L-4)x_4, \tag{32}
$$

always holds.

This ends the analyses on three-photon case.

SUPPLEMENTARY NOTE 4 - SECURITY PROOF IN THE FOUR-PHOTON CASE

Alice randomly prepares the four-photon state

$$
|\psi\rangle = \sum_{i=1}^{L} |iiii\rangle + \sum_{i<j} |iijj\rangle + (-1)^{k_i+k_j}(|ijjj\rangle + |iiij\rangle + \sum_{n\neq i,j} |ijnn\rangle) + \sum_{i<j<l<m} (-1)^{k_i+k_j+k_l+k_m}|ijlm\rangle, \tag{33}
$$

where we treat the efficiencies as part of quantum state, e.g., $a|ijlm\rangle$ is simply denoted by $|ijlm\rangle$. Similar to the single photon case, Eve's general collective attack in four-photon case can be given by:

$$U_{\text{Eve}}|ijlm\rangle|e_{00000}\rangle = \sum_{t=1}^{L} c_{ijlmt}|t\rangle\big|e_{ijlmt}\big\rangle. \tag{34}$$

When Bob projects the incoming single photon states into $(|a\rangle \pm |b\rangle)/\sqrt{2}$ successfully, the evolution of quantum state will be

$$\begin{aligned}
|\psi\rangle|e_{00000}\rangle \longrightarrow & (\tilde{c}_a|a\rangle + \tilde{c}_b|b\rangle) + (-1)^{k_a+k_b}(\tilde{c}_{aba}|a\rangle + \tilde{c}_{abb}|b\rangle) \\
& + \sum_{i\neq a,b} (-1)^{k_i}(\tilde{c}_{iaa}|a\rangle + \tilde{c}_{iab}|b\rangle + (-1)^{k_a+k_b}(\tilde{c}_{iba}|a\rangle + \tilde{c}_{ibb}|b\rangle)) \\
& + \sum_{i,j\neq a,b} (-1)^{k_i+k_j}((\tilde{c}_{ija}|a\rangle + \tilde{c}_{ijb}|b\rangle) + (-1)^{k_a+k_b}(\tilde{c}_{ijaba}|a\rangle + \tilde{c}_{ijabb}|b\rangle)) \\
& + \sum_{i,j,l\neq a,b} (-1)^{k_i+k_j+k_l}(\tilde{c}_{ijlaa}|a\rangle + \tilde{c}_{ijlab}|b\rangle + (-1)^{k_a+k_b}(\tilde{c}_{ijlba}|a\rangle + \tilde{c}_{ijlbb}|b\rangle)) \\
& + \sum_{i,j,l,m\neq a,b} (-1)^{k_i+k_j+k_l+k_m}(\tilde{c}_{ijlma}|a\rangle + \tilde{c}_{ijlmb}|b\rangle),
\end{aligned} \tag{35}$$

where, $\tilde{c}_t \triangleq \sum_{i=1}^{L} \tilde{c}_{iiiit} + \sum_{i<j} \tilde{c}_{iijjt}$,$\tilde{c}_{ijt} \triangleq \tilde{c}_{ijjjt} + \tilde{c}_{iiijt} + \sum_{n\neq i,j} \tilde{c}_{ijnnt}$,and $\tilde{c}_{ijlmt} = c_{ijlmt}\big|e_{ijlmt}\big\rangle$. Clearly, each row has the same pattern with evolution of two-photon case. Based on similar techniques used in last three subsections, we have

$$I_{\text{AE}} \leqslant \frac{\varphi((L-1)x_1, x_2) + \varphi((L-2)x_2, 2x_3) + \varphi((L-3)x_3, 3x_4) + \varphi((L-4)x_4, 4x_5)}{(L-1)(x_1 + x_2 + x_3 + x_4 + x_5)}, \tag{36}$$

where,

$$\begin{aligned}
x_1 &\triangleq \sum_i \big|\tilde{c}_i\big|^2, \\
x_2 &\triangleq \sum_{a<b} \big|\tilde{c}_{aba}\big|^2 + \big|\tilde{c}_{abb}\big|^2, \\
x_3 &\triangleq \sum_{a<b} \sum_{i\neq a,b} \big|\tilde{c}_{abi}\big|^2, \\
x_4 &\triangleq \sum_{i<j<l<m} c_{ijlmi}^2 + c_{ijlmj}^2 + c_{ijlml}^2 + c_{ijlmm}^2, \\
x_5 &\triangleq \sum_{i<j<l<m} \sum_{t\neq i,j,l,m} c_{ijlmt}^2.
\end{aligned} \tag{37}$$

And these parameters are constrained by the error rate E,

$$E \geqslant \frac{(\sqrt{(L-1)x_1} - \sqrt{x_2})^2 + (\sqrt{(L-3)x_3} - \sqrt{3x_4})^2 + (L-5)x_5/2}{(L-1)(x_1 + x_2 + x_3 + x_4 + x_5)}. \tag{38}$$

This ends the analyses on four-photon case.

SUPPLEMENTARY NOTE 5 - SECURITY PROOF IN THE ODD PHOTON-NUMBER CASE

Alice randomly prepares an encoding state like before, but the photon-number N is an odd number and $L \geqslant N+1$. It is clear that her encoding state has the form

$$\begin{aligned}|\psi\rangle =&\sum_{i_1}(-1)^{k_{i_1}}|i_1\rangle + \sum_{i_1<i_2<i_3}(-1)^{k_{i_1}+k_{i_2}+k_{i_3}}|i_1i_2i_3\rangle + \sum_{i_1<i_2<i_3<i_4<i_5}(-1)^{k_{i_1}+k_{i_2}+k_{i_3}+k_{i_4}+k_{i_5}}|i_1i_2i_3i_4i_5\rangle \\ &+ ... + \sum_{i_1<i_2<i_3<...<N}(-1)^{k_{i_1}+k_{i_2}+k_{i_3}+...+k_{i_N}}|i_1i_2i_3...i_N\rangle,\end{aligned} \tag{39}$$

i.e., it consists of n-phase ($n = 1, 3, 5, ..., N$) state, denoted by $|i_1i_2..i_n\rangle$. For example, $|i_1i_2...i_n\rangle$ represents that the photon number in time-bins i_1, i_2,..., and i_n must be odd, while the photon numbers in all other time-bins must be even. Eve's general collective attack in this case can be given by:

$$U_{\text{Eve}}|i_1i_2..i_n\rangle|e_{initial}\rangle = \sum_{t=1}^{L} c_{i_1i_2..i_nt}|t\rangle|e_{i_1i_2..i_nt}\rangle \triangleq \sum_{t=1}^{L}\tilde{c}_{i_1i_2..i_nt}|t\rangle. \tag{40}$$

When Bob projects the incoming single photon states into $(|a\rangle \pm |b\rangle)/\sqrt{2}$ successfully, the evolution of quantum state will be

$$\begin{aligned}|\psi\rangle|e_{initial}\rangle \longrightarrow &(-1)^{k_a}(\tilde{c}_{aa}+\tilde{c}_{ab}) + (-1)^{k_b}(\tilde{c}_{ba}+\tilde{c}_{bb}) \\ &+ \sum_{i_1\neq a,b}(-1)^{i_1}(\tilde{c}_{i_1a}|a\rangle + \tilde{c}_{i_1b}|b\rangle) + (-1)^{k_a+k_b}(\tilde{c}_{i_1aba}|a\rangle + \tilde{c}_{i_1abb}|b\rangle) \\ &+ \sum_{i_1i_2\neq a,b}(-1)^{k_{i_1}+k_{i_2}}((\tilde{c}_{i_1i_2aa}|a\rangle + \tilde{c}_{i_1i_2ab}|b\rangle) + (-1)^{k_a+k_b}(\tilde{c}_{i_1i_2ba}|a\rangle + \tilde{c}_{i_1i_2bb}|b\rangle)) \\ &+ ... \\ &+ \sum_{i_1...i_{N-2}\neq a,b}(-1)^{k_{i_1}+...+k_{i_{N-2}}}((\tilde{c}_{i_1...i_{N-2}a}|a\rangle + \tilde{c}_{i_1...i_{N-2}b}|b\rangle) \\ &\qquad + (-1)^{k_a+k_b}(\tilde{c}_{i_1...i_{N-2}aba}|a\rangle + \tilde{c}_{i_1...i_{N-2}abb}|b\rangle)) \\ &+ \sum_{i_1...i_{N-1}\neq a,b}(-1)^{k_{i_1}+k_{i_2}+..+k_{i_{N-1}}}((\tilde{c}_{i_1...i_{N-1}aa}|a\rangle + \tilde{c}_{i_1...i_{N-1}ab}|b\rangle) \\ &\qquad + (-1)^{k_a+k_b}(\tilde{c}_{i_1...i_{N-1}ba}|a\rangle + \tilde{c}_{i_1...i_{N-1}bb}|b\rangle)) \\ &+ \sum_{i_1...i_N\neq a,b}(-1)^{k_{i_1}+...+k_{i_N}}(\tilde{c}_{i_1...i_Na}|a\rangle + \tilde{c}_{i_1...i_Nb}|b\rangle).\end{aligned} \tag{41}$$

Evidently, for each summation we can calculate Eve's information. Specifically, for the summations with the global phase $(-1)^{k_{i_1}+..+k_{i_n}}$ and n is odd, we obtain

$$Q^{(a,b)}I_{\text{AE}}^{(a,b)} \leqslant \sum_{i_1...i_n\neq a,b}\varphi(\left|\tilde{c}_{i_1...i_na}\right|^2 + \left|\tilde{c}_{i_1...i_nb}\right|^2, \left|\tilde{c}_{i_1...i_naba}\right|^2 + \left|\tilde{c}^2_{i_1...+i_nabb}\right|^2). \tag{42}$$

For the summations with the global phase $(-1)^{k_{i_1}+..+k_{i_n}}$ and n is even, we obtain

$$Q^{(a,b)}I_{\text{AE}}^{(a,b)} \leqslant \sum_{i_1...i_n\neq a,b}\varphi(\left|\tilde{c}_{i_1...i_naa}\right|^2 + \left|\tilde{c}_{i_1...i_nbb}\right|^2, \left|\tilde{c}_{i_1...i_nab}\right|^2 + \left|\tilde{c}^2_{i_1...+i_nba}\right|^2). \tag{43}$$

Noting the following mathematical identities

$$
\begin{aligned}
&\sum_{a<b} \left|\tilde{c}_{aa}\right|^2 + \left|\tilde{c}_{bb}\right|^2 = (L-1)\sum_{i} \left|\tilde{c}_{ii}\right|^2, \\
&\sum_{a<b} \left|\tilde{c}_{ab}\right|^2 + \left|\tilde{c}_{ba}\right|^2 = \sum_{i\neq j} \left|\tilde{c}_{ij}\right|^2, \\
&\sum_{a<b} \sum_{i_1\ldots i_n\neq a,b} \left|\tilde{c}_{i_1\ldots i_n a}\right|^2 + \left|\tilde{c}_{i_1\ldots i_n b}\right|^2 = (L-n-1)\sum_{i_1\ldots i_n} \sum_{t\neq i_1\ldots i_n} \left|\tilde{c}_{i_1\ldots i_n t}\right|^2, \\
&\sum_{a<b} \sum_{i_1\ldots i_n\neq a,b} \left|\tilde{c}_{i_1\ldots i_n aa}\right|^2 + \left|\tilde{c}_{i_1\ldots i_n bb}\right|^2 = (L-n-1)\sum_{i_1\ldots i_{n+1}} \sum_{t=i_1}^{i_{n+1}} c_{i_1\ldots i_{n+1} t}, \\
&\sum_{a<b} \sum_{i_1\ldots i_{n+1}\neq a,b} \left|\tilde{c}_{i_1\ldots i_n ba}\right|^2 + \left|\tilde{c}_{i_1\ldots i_n ab}\right|^2 = (n+1)\sum_{i_1\ldots i_{n+1}} \sum_{t\neq i_1\ldots i_{n+1}} c_{i_1\ldots i_{n+1} t}, \\
&\sum_{a<b} \sum_{i_1\ldots i_n\neq a,b} \left|\tilde{c}_{i_1\ldots i_n aba}\right|^2 + \left|\tilde{c}_{i_1\ldots i_n abb}\right|^2 = (n+1)\sum_{i_1\ldots i_{n+2}} \sum_{t=i_1}^{i_{n+2}} c_{i_1\ldots i_{n+2} t}.
\end{aligned} \tag{44}
$$

And define

$$
\begin{aligned}
x_1 &\triangleq \sum_{i} \left|\tilde{c}_{ii}\right|^2, \\
x_2 &\triangleq \sum_{i\neq j} \left|\tilde{c}_{ij}\right|^2, \\
x_n &\triangleq \sum_{i_1\ldots i_n} \sum_{t=i_1}^{i_n} \left|\tilde{c}_{i_1\ldots i_n t}\right|^2, \\
x_{n+1} &\triangleq \sum_{i_1\ldots i_n} \sum_{t\neq i_1\ldots i_n} \left|\tilde{c}_{i_1\ldots i_n t}\right|^2.
\end{aligned} \tag{45}
$$

Combining Eqs.(42), (43), (44) and (45), we have

$$
\sum_{a<b} Q^{(a,b)} I_{\mathrm{AE}}^{(a,b)} \leqslant \varphi((L-1)x_1, x_2) + \varphi((L-2)x_2, 2x_3) + \varphi((L-3)x_3, 3x_4) + \ldots + \varphi((L-N)x_N, Nx_{N+1}). \tag{46}
$$

Besides, with Eqs.(39), (40) and (45), it's easy to verify $\sum_{a<b} Q^{(a,b)} = (L-1)(x_1 + x_2 + \ldots + x_{N+1})$. In conclusion, Eve's information is

$$
I_{\mathrm{AE}} = \frac{\sum_{a<b} Q^{(a,b)} I_{\mathrm{AE}}^{(a,b)}}{\sum_{a<b} Q^{(a,b)}} \leqslant \frac{\sum_{n=1}^{N} \varphi((L-n)x_n, nx_{n+1})}{(L-1)\sum_{n=1}^{N+1} x_n}. \tag{47}
$$

Through calculating the probabilities of error-key events corresponds to the n-th (n is even) row of Eq.(41), we obtain that the error rate E must satisfy

$$
E \geqslant \frac{\sum_{n\geqslant 1}^{(N-1)/2} (\sqrt{(L-2n)x_{2n}} - \sqrt{2nx_{2n+1}})^2 + (L-N-1)x_{N+1}/2}{(L-1)\sum_{n=1}^{N+1} x_n}. \tag{48}
$$

This ends the security proof for odd photon-number case.

SUPPLEMENTARY NOTE 6 - SECURITY PROOF IN THE EVEN PHOTON-NUMBER CASE

Alice randomly prepares an encoding state like before, but the photon-number N is an even number and $L \geqslant N+1$. It is clear that her encoding state has the form

$$
\begin{aligned}
|\psi\rangle = & |i_0\rangle + \sum_{i_1<i_2}(-1)^{k_{i_1}+k_{i_2}}|i_1 i_2\rangle + \sum_{i_1<i_2<i_3<i_4}(-1)^{k_{i_1}+k_{i_2}+k_{i_3}+k_{i_4}}|i_1 i_2 i_3 i_4\rangle \\
& + ... + \sum_{i_1<i_2<i_3<...<i_N}(-1)^{k_{i_1}+k_{i_2}+k_{i_3}+...+k_{i_N}}|i_1 i_2 i_3 ... i_N\rangle,
\end{aligned}
\tag{49}
$$

i.e., it consists of n-phase ($n = 0, 2, 4, ..., N$) state, denoted by $|i_1 i_2..i_n\rangle$. For example, $|i_1 i_2 ... i_n\rangle$ represents that the photon number in time-bins i_1, i_2,..., and i_n must be odd, while the photon numbers in all other time-bins must be even. Eve's general collective attack in this case can be given by:

$$
U_{\text{Eve}}|i_1 i_2..i_n\rangle|e_{initial}\rangle = \sum_{t=1}^{L} c_{i_1 i_2..i_n t}|t\rangle|e_{i_1 i_2..i_n t}\rangle \triangleq \sum_{t=1}^{L} \tilde{c}_{i_1 i_2..i_n t}|t\rangle. \tag{50}
$$

When Bob projects the incoming single photon states into $(|a\rangle \pm |b\rangle)/\sqrt{2}$ successfully, the evolution of quantum state will be

$$
\begin{aligned}
|\psi\rangle|e_{initial}\rangle \longrightarrow & (\tilde{c}_{i_0 a}|a\rangle + \tilde{c}_{i_0 b}|b\rangle) + (-1)^{k_a+k_b}(\tilde{c}_{aba}|a\rangle + \tilde{c}_{abb}|b\rangle) \\
& + \sum_{i_1 \neq a,b}(-1)^{i_1}(\tilde{c}_{i_1 aa}|a\rangle + \tilde{c}_{i_1 ab}|b\rangle) + (-1)^{k_a+k_b}(\tilde{c}_{i_1 ba}|a\rangle + \tilde{c}_{i_1 bb}|b\rangle) \\
& + \sum_{i_1 i_2 \neq a,b}(-1)^{k_{i_1}+k_{i_2}}((\tilde{c}_{i_1 i_2 a}|a\rangle + \tilde{c}_{i_1 i_2 b}|b\rangle) + (-1)^{k_a+k_b}(\tilde{c}_{i_1 i_2 aba}|a\rangle + \tilde{c}_{i_1 i_2 abb}|b\rangle)) \\
& + ... \\
& + \sum_{i_1 ... i_{N-2} \neq a,b}(-1)^{k_{i_1}+...+k_{i_{N-2}}}((\tilde{c}_{i_1 ... i_{N-2} a}|a\rangle + \tilde{c}_{i_1 ... i_{N-2} b}|b\rangle) \\
& \qquad + (-1)^{k_a+k_b}(\tilde{c}_{i_1 ... i_{N-2} aba}|a\rangle + \tilde{c}_{i_1 ... i_{N-2} abb}|b\rangle)) \\
& + \sum_{i_1 ... i_{N-1} \neq a,b}(-1)^{k_{i_1}+k_{i_2}+..+k_{i_{N-1}}}((\tilde{c}_{i_1 ... i_{N-1} aa}|a\rangle + \tilde{c}_{i_1 ... i_{N-1} ab}|b\rangle) \\
& \qquad + (-1)^{k_a+k_b}(\tilde{c}_{i_1 ... i_{N-1} ba}|a\rangle + \tilde{c}_{i_1 ... i_{N-1} bb}|b\rangle)) \\
& + \sum_{i_1 ... i_N \neq a,b}(-1)^{k_{i_1}+...+k_{i_N}}(\tilde{c}_{i_1 ... i_N a}|a\rangle + \tilde{c}_{i_1 ... i_N b}|b\rangle).
\end{aligned}
\tag{51}
$$

Evidently, for each summation we can calculate Eve's information. Specifically, for the summations with the global phase $(-1)^{k_{i_1}+..+k_{i_n}}$ and n is even, we obtain

$$
\begin{aligned}
Q^{(a,b)} I_{\text{AE}}^{(a,b)} \leqslant & \varphi(|\tilde{c}_{i_0 a}|^2 + |\tilde{c}_{i_0 b}|^2, |\tilde{c}_{aba}|^2 + |\tilde{c}^2_{abb}|^2) \\
& \sum_{i_1 ... i_n \neq a,b} \varphi(|\tilde{c}_{i_1 ... i_n a}|^2 + |\tilde{c}_{i_1 ... i_n b}|^2, |\tilde{c}_{i_1 ... i_n aba}|^2 + |\tilde{c}^2_{i_1 ... + i_n abb}|^2).
\end{aligned}
\tag{52}
$$

For the summations with the global phase $(-1)^{k_{i_1}+..+k_{i_n}}$ and n is odd, we obtain

$$
Q^{(a,b)} I_{\text{AE}}^{(a,b)} \leqslant \sum_{i_1 ... i_n \neq a,b} \varphi(|\tilde{c}_{i_1 ... i_n aa}|^2 + |\tilde{c}_{i_1 ... i_n bb}|^2, |\tilde{c}_{i_1 ... i_n ab}|^2 + |\tilde{c}^2_{i_1 ... + i_n ba}|^2). \tag{53}
$$

Noting the following mathematical identities

$$
\begin{aligned}
&\sum_{a<b} \left|\tilde{c}_{i_0 a}\right|^2 + \left|\tilde{c}_{i_0 b}\right|^2 = (L-1)\sum_{j} \left|\tilde{c}_{i_0 j}\right|^2, \\
&\sum_{a<b} \left|\tilde{c}_{aba}\right|^2 + \left|\tilde{c}_{abb}\right|^2 = \sum_{i_1<i_2} \left|\tilde{c}_{i_1 i_2 i_1}\right|^2 + \left|\tilde{c}_{i_1 i_2 i_2}\right|^2, \\
&\sum_{a<b} \sum_{i_1 \dots i_n \neq a,b} \left|\tilde{c}_{i_1 \dots i_n a}\right|^2 + \left|\tilde{c}_{i_1 \dots i_n b}\right|^2 = (L-n-1) \sum_{i_1 \dots i_n} \sum_{t \neq i_1 \dots i_n} \left|\tilde{c}_{i_1 \dots i_n t}\right|^2, \\
&\sum_{a<b} \sum_{i_1 \dots i_n \neq a,b} \left|\tilde{c}_{i_1 \dots i_n aa}\right|^2 + \left|\tilde{c}_{i_1 \dots i_n bb}\right|^2 = (L-n-1) \sum_{i_1 \dots i_{n+1}} \sum_{t=i_1}^{i_{n+1}} c_{i_1 \dots i_{n+1} t}, \\
&\sum_{a<b} \sum_{i_1 \dots i_{n+1} \neq a,b} \left|\tilde{c}_{i_1 \dots i_n ba}\right|^2 + \left|\tilde{c}_{i_1 \dots i_n ab}\right|^2 = (n+1) \sum_{i_1 \dots i_{n+1}} \sum_{t \neq i_1 \dots i_{n+1}} c_{i_1 \dots i_{n+1} t}, \\
&\sum_{a<b} \sum_{i_1 \dots i_n \neq a,b} \left|\tilde{c}_{i_1 \dots i_n aba}\right|^2 + \left|\tilde{c}_{i_1 \dots i_n abb}\right|^2 = (n+1) \sum_{i_1 \dots i_{n+2}} \sum_{t=i_1}^{i_{n+2}} c_{i_1 \dots i_{n+2} t}.
\end{aligned} \tag{54}
$$

And define

$$
\begin{aligned}
x_1 &\triangleq \sum_{j} \left|\tilde{c}_{i_0 j}\right|^2, \\
x_2 &\triangleq \sum_{i<j} \left|\tilde{c}_{iji}\right|^2 + \left|\tilde{c}_{ijj}\right|^2, \\
x_n &\triangleq \sum_{i_1 \dots i_n} \sum_{t=i_1}^{i_n} \left|\tilde{c}_{i_1 \dots i_n t}\right|^2, \\
x_{n+1} &\triangleq \sum_{i_1 \dots i_n} \sum_{t \neq i_1 \dots i_n} \left|\tilde{c}_{i_1 \dots i_n t}\right|^2.
\end{aligned} \tag{55}
$$

Combining Eqs.(52), (53), (54) and (55), we have

$$
\sum_{a<b} Q^{(a,b)} I_{\mathrm{AE}}^{(a,b)} \leqslant \varphi((L-1)x_1, x_2) + \varphi((L-2)x_2, 2x_3) + \varphi((L-3)x_3, 3x_4) + \dots + \varphi((L-N)x_N, N x_{N+1}). \tag{56}
$$

Besides, with Eqs.(49), (50) and (55), it's easy to verify $\sum_{a<b} Q^{(a,b)} = (L-1)(x_1 + x_2 + \dots + x_{N+1})$. In conclusion, Eve's information is

$$
I_{\mathrm{AE}} = \frac{\sum_{a<b} Q^{(a,b)} I_{\mathrm{AE}}^{(a,b)}}{\sum_{a<b} Q^{(a,b)}} \leqslant \frac{\sum_{n=1}^{N} \varphi((L-n)x_n, n x_{n+1})}{(L-1)\sum_{n=1}^{N+1} x_n}. \tag{57}
$$

Through calculating the probabilities of error-key events corresponds to the n-th (n is odd) row of Eq.(51), we obtain that the error rate E must satisfy

$$
E \geqslant \frac{\sum_{n\geqslant 1}^{N/2} (\sqrt{(L-2n+1)x_{2n-1}} - \sqrt{(2n-1)x_{2n}})^2 + (L-N-1)x_{N+1}/2}{(L-1)\sum_{n=1}^{N+1} x_n}. \tag{58}
$$

This ends the security proof for even photon-number case.

SUPPLEMENTARY NOTE 7 - CONCLUSION IN GENERAL N-PHOTON CASE

We summarize and simplify the results given by the even photon-number and odd photon-number cases here. For a RRDPS protocol with N photon-number source, packet size L and $L \geqslant N+1$, Eve's information can by bounded by

$$I_{\mathrm{AE}} \leqslant \frac{\sum_{n=1}^{N} \varphi((L-n)x_n, nx_{n+1})}{L-1}, \tag{59}$$

where, $\varphi(x,y) = -x\log_2 x - y\log_2 y + (x+y)\log_2(x+y)$, and non-negative real parameters x_i satisfying $\sum_{i=1}^{N+1} x_i = 1$. If Alice and Bob make sure that their error rate is E, then the parameters will also satisfy that:

$$\begin{aligned}
&\text{if } N \text{ is odd,}\\
&E \geqslant \frac{\sum_{n\geqslant 1}^{(N-1)/2}(\sqrt{(L-2n)x_{2n}} - \sqrt{2nx_{2n+1}})^2 + (L-N-1)x_{N+1}/2}{L-1};\\
&\text{if } N \text{ is even,}\\
&E \geqslant \frac{\sum_{n\geqslant 1}^{N/2}(\sqrt{(L-2n+1)x_{2n-1}} - \sqrt{(2n-1)x_{2n}})^2 + (L-N-1)x_{N+1}/2}{L-1}.
\end{aligned} \tag{60}$$

Base one above results, a corollary is straightforward which is: for any $N < L-1$, $I_{\mathrm{AE}} < 1$ holds. Lets prove this corollary by reduction to absurdity. We consider $I_{\mathrm{AE}} = 1$ in case of $N < L-1$. According to the property of $\varphi(x,y)$ function,

$$\begin{aligned}
1 = I_{\mathrm{AE}} &\leqslant \frac{\sum_{n=1}^{N} \varphi((L-n)x_n, nx_{n+1})}{L-1} \leqslant \frac{\sum_{n=1}^{N}(L-n)x_n + nx_{n+1}}{L-1} = \frac{\sum_{n=1}^{N}(L-1)x_n + Nx_{N+1}}{L-1}\\
&= 1 - x_{N+1} + \frac{N}{L-1}x_{N+1} = 1 + \frac{N-(L-1)}{L-1}x_{N+1}.
\end{aligned} \tag{61}$$

Evidently, this suggests that $x_{N+1} = 0$, which leads to $\varphi((L-N)x_N, x_{N+1}) = 0$. Then Eq.(61) is rewritten as

$$\begin{aligned}
1 = I_{\mathrm{AE}} &\leqslant \frac{\sum_{n=1}^{N-1} \varphi((L-n)x_n, nx_{n+1})}{L-1} \leqslant \frac{\sum_{n=1}^{N-1}(L-1)x_n + Nx_N}{L-1}\\
&= 1 - x_N + \frac{N-1}{L-1}x_N = 1 + \frac{N-1-(L-1)}{L-1}x_N,
\end{aligned} \tag{62}$$

which implies $x_N = 0$. Through repeating above arguments for N times, we obtain that $x_n = 0 (n = 2, 3, 4..)$ and $I_{\mathrm{AE}} = 0$, which conflicts with $I_{\mathrm{AE}} = 1$. This ends the proof of this corollary.

SUPPLEMENTARY REFERENCES

[1] Holevo, A. S. Some estimates of the information transmitted by quantum communication channels. *Engl. Transl. Probl. Inf. Trans.* **9**, 177-183 (1973).
